# Influence of hot‐smoking on the stability of fresh and frozen–thawed deep‐skinned Atlantic mackerel fillets during cold storage

**DOI:** 10.1002/fsn3.4132

**Published:** 2024-04-08

**Authors:** Carina Mascarenhas Fernandes, Hildur Inga Sveinsdóttir, Tumi Tómasson, Sigurjón Arason, María Gudjónsdóttir

**Affiliations:** ^1^ Faculty of Food Science and Nutrition University of Iceland Reykjavík Iceland; ^2^ Faculty of Sciences and Technology University of Cape Verde Praia, Santiago Cape Verde; ^3^ Matís Ohf./Icelandic Food and Biotech R&D Reykjavík Iceland; ^4^ UNESCO GRÓ—Fisheries Training Programme Hafnarfjorður Iceland

**Keywords:** Atlantic mackerel, deep‐skinned, fresh, frozen, hot‐smoking, quality

## Abstract

Atlantic mackerel (*Scomber scombrus*) caught during the summer months in Icelandic waters after intensive feeding is rich in lipids and, thus, sensitive to lipid degradation. Recent studies have led to improved cooling and handling on board, ensuring high‐quality raw material. However, studies on the development of high‐quality products for human consumption are lacking. The study aimed to investigate the effects of hot‐smoking on the physicochemical, microbial, and sensory quality of deep‐skinned Atlantic mackerel fillets during chilled storage (1 ± 0.6°C). In addition, the quality of smoked mackerel from frozen–thawed fillets (9 months at −25 ± 1.8°C) was compared to that of fresh‐smoked fillets to evaluate the possibility of the industry being able to provide smoked fillets throughout the year, despite the short fishing season. Brining and hot‐smoking reduced total viable counts and inactivated *Listeria monocytogenes*. Hot‐smoking positively affected the sensory attributes of the fillets and sensory quality was largely maintained for at least 21 days of chilled storage. Although slightly lower sensory and texture scores were obtained for frozen–thawed smoked fillets, they remained within acceptable limits throughout the period of cold storage. The shelf‐life of smoked Atlantic mackerel deep‐skinned fillets stored at 1°C is, therefore, assessed to be at least 21 days. Well‐fed Atlantic mackerel is suitable for developing high‐quality and stable smoked fillet products from both fresh and frozen–thawed raw materials.

## Graphical abstract

This study examined the impact of hot‐smoking on the quality of deep‐skinned Atlantic mackerel fillets that are prone to autolysis and lipid oxidation due to intensive feeding in Icelandic waters during summer. Hot‐smoking improved sensory attributes and microbial safety of the fillets, allowing them to be stored for at least 21 days at 1°C. Well‐fed Atlantic mackerel can be used to create high‐quality smoked fillets from both fresh and frozen‐thawed raw materials, potentially extending the availability of such products beyond the short fishing season.

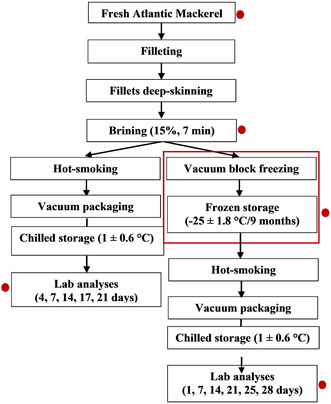

## 1 INTRODUCTION

Atlantic mackerel (*Scomber scombrus*) is among the most important commercial pelagic fish species in Europe (EUMOFA, 2018), but, in Iceland, it has until recently primarily been processed for fishmeal and fish oil. The fishing season is very short since the mackerel typically only spends a few weeks in Icelandic waters during feeding migration in late summer (Romotowska, Karlsdóttir, et al., 2016a; Sveinsdóttir et al., 2020). During this time, the lipid content reaches up to 30 g of lipid/100 g of sample. This poses challenges in the handling and processing of mackerel as it becomes highly susceptible to lipid deterioration, which can cause quality and stability changes (Romotowska, Karlsdóttir, et al., 2016a). Lipid oxidation can lead to off‐flavors, alterations in color and texture, and nutrient loss (Ozogul & Balikci, 2013). Oxidation of muscle lipids involves the peroxidation of unsaturated fatty acids, particularly polyunsaturated fatty acids (PUFAs), which are abundant in Atlantic mackerel.

During hot‐smoking, the fish is smoked at an appropriate combination of temperatures and time that ensures the destruction or inactivation of bacterial pathogens, parasites, and spores (FAO, 2013). However, the chemical, physical, and nutritional composition of the fish undergoes complex changes, such as flavor development, texture and color modifications, and reduction in moisture content (Abraha et al., 2018). The presence of volatile aldehydes, which are responsible for off‐flavors in smoked salmon spoiled by bacteria, has been attested by some researchers, but their origin (lipid oxidation, wood smoke, or microbial growth) could not be confirmed (Varlet et al., 2007). Rana et al. (2021) stated that the sensory attributes of fish and fish products can be negatively affected by metabolites released through spoilage mechanisms, which can shorten shelf‐life. If heating during processing becomes excessive, protein denaturation may occur and nutrients may be lost (Abraha et al., 2018).

Recent studies have suggested that rapid cooling and improved handling of the catch can overcome challenges in processing, allowing for the production of nutritious products for human consumption (Sveinsdóttir et al., 2020). Removing the skin and the dark muscle through deep‐skinning has been shown to increase the stability of frozen Atlantic mackerel fillets (Dang et al., 2017, 2018; Sveinsdóttir et al., 2020). These advances make further value‐adding processing possible.

Recently, several studies have been carried out on many different fish preservation techniques (Cheng et al., 2023a, 2023b; Kumar et al., 2022; Lu et al., 2022). Over the years, many studies aimed specifically at mackerel species have focused on the effects of smoking, each using different smoking processes, treatments, packaging methods, and technologies (Baten et al., 2020; Goulas & Kontominas, 2005; Huang et al., 2019; Kolodziejska et al., 2002; Ozogul & Balikci, 2013; Stolyhwo et al., 2006). However, to the best of the authors' knowledge, the particular combination of hot‐smoking and vacuum‐packaging applied to fresh and frozen deep‐skinned fillets from well‐fed Atlantic mackerel has not been addressed. A shelf‐life study was therefore conducted, assessing the effects of hot‐smoking on the safety and stability of the fillets during chilled storage, and to explore whether hot‐smoking could be applied to both fresh and frozen–thawed fillets. Such an approach could increase flexibility and value addition in the processing of this highly seasonal raw material.

## 2 MATERIALS AND METHODS

### 2.1 Chemicals and reagents

The chemicals used for the laboratory analyses were of analytical grade and were purchased from Fluka (Bush, Switzerland) and Sigma‐Aldrich (Steinheim, Germany/St. Louis, MO, USA).

### 2.2 Raw material and sampling schedule

Atlantic mackerel were caught in Icelandic waters at the end of August 2019 using a pelagic trawl. The fish was chilled to −1.5°C on board using refrigerated sea water (RSW). The batch (200 tons) of fresh whole mackerel 40–50 cm long, weighing 300–500 g, was collected from a single haul that arrived at the processing facilities of the fishing company approximately 48 h after catching. The fresh fish was filleted mechanically (VMK11‐M120 Arenco VMK, Sweden) and deep‐skinned (approximately 4 mm cutting depth) with a Trio skinning machine (FDS 105‐T, Trio Fish Processing Machinery AS). The subcutaneous dark muscle was completely removed, retaining only the light muscle and a small portion of the medial dark muscle. All the deep‐skinned fillets, which were on average approximately 20 cm long and 80 g, were then brined in 15% sodium chloride (NaCl) (100 g/L) for 7 min. Around 50 fillets were then hot‐smoked, vacuum‐packed, iced, placed in a styrofoam box, and kept under refrigerated conditions (1 ± 0.6°C) for 2 days before being transported to the research facilities. Another sample of fillets was frozen in 16 kg semi‐vacuum boxes with 2% brine added to make up 4% of the block weight (Non‐Pressure Plate Freezer—SKAGINN 3×) at −45°C for 5 h, resulting in an average core temperature of −25°C. About 10 boxes were then labeled, loaded on pallets, and stored at −25 ± 1.5°C. Later, the frozen boxes were transported to the research station in a container truck (−25°C) and kept for 9 months at −25 ± 1.8°C before being thawed and hot‐smoked.

### 2.3 Hot‐smoking and packaging

All fillets were rinsed in tap water and spread out on drying racks to allow the brine to drain for about 30 min at ambient temperature before being placed inside an electrical smoker (Smoker UW‐150, Borniak, Poland) with an automatic temperature controller. The smoke inside the kiln was generated from beechwood sawdust (Dansk Træmel, GL Esbjerg, Denmark). The kiln was preheated for 1 h, before smoking, which was carried out in three stages: (1) a preliminary drying stage at 30°C for 30 min; (2) a partial cooking and smoking at 50°C for 30 min; and (3) a final smoking and cooking stage at 80°C for 45 min for fresh fillets and 90 min for frozen–thawed fillets, until a consistent brown color and a firm muscle were achieved. After smoking, the fillets were left to cool at room temperature for about 30 min, before being placed in 75‐μm thick low‐density polyethylene (LDPE) plastic bags (Kivo, Volendam, Netherlands) and vacuum‐packed (ATM Vacuum Packer‐Packman XL), 5–6 fillets in each bag. The samples were then placed in a styrofoam box and kept at 1 ± 0.6°C for up to 28 days until sampled for analysis.

### 2.4 Sampling and sample preparation

**FIGURE 1:**
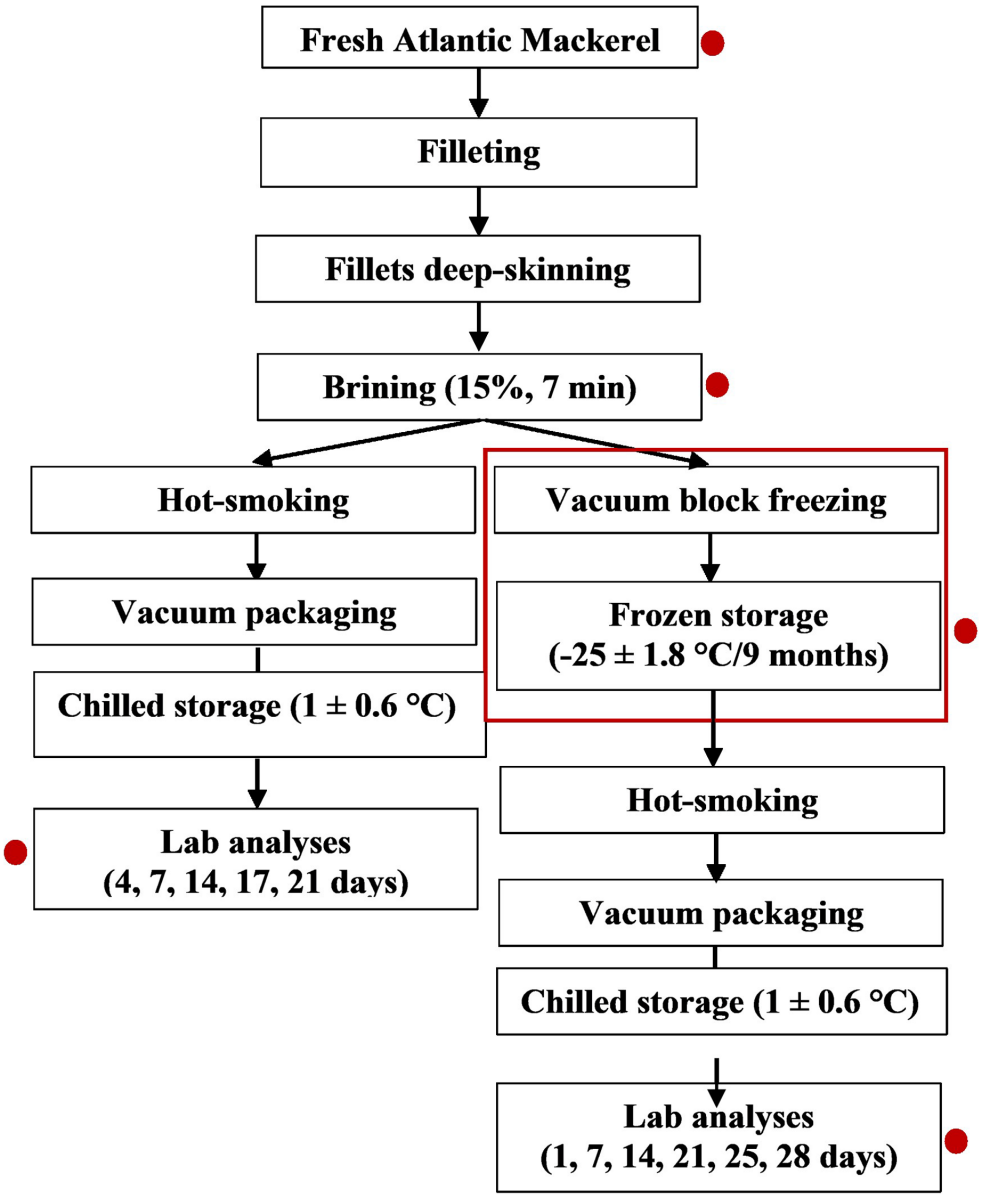
Experimental design and sampling of deep‐skinned Atlantic mackerel fillets. Red dots indicate sampling times.

Physicochemical and microbiological analyses were carried out on fresh fillets, on frozen fillets after thawing (24 h at 1 ± 1.6°C), and after brining and hot‐smoking of both fresh and frozen–thawed fillets. Quality changes were monitored throughout 28 days of chilled storage (1 ± 1.6°C) (Figure 1). On each sampling occasion, six fillets were taken for the physicochemical analyses and divided into three sets of two fillets that were minced, placed in sterile plastic containers, and labeled before physicochemical analyses. To conduct microbiological assessments, five fillets were pooled and minced. Sensory evaluation of smoked fillets was carried out on days 4, 7, 14, 17, and 21 of cold storage for the fresh‐smoked fillets, and on days 1, 7, 14, 21, 25, and 28 for the frozen–thawed smoked fillets. The fresh‐smoked fillets were only analyzed from day 4 since the transport from the processing facilities in the East of Iceland to the laboratory facilities in Reykjavík took 2 days. While evaluating the frozen‐smoked fillets, a decision was taken to extend the trial by 1 week, adding sampling on day 25 and day 28 of chilled storage. The analytical methods used are described in the following sections.

### 2.5 Microbiological analyses

To assess *Listeria monocytogenes*, 25 g of the minced sample was used, while 20 g was utilized for determining total aerobic viable counts (TVC). Dilutions, made by a factor of 10 from the initial amount, were pipetted onto two separate sets of plates to perform duplicate assessments of each sample. *L. monocytogenes* isolation and identification in the fish muscle was performed, as described by the Nordic‐Baltic Committee on Food Analysis—NMKL (2010), and expressed as either detected or not detected. The results of the TVC were expressed as log_10_ of colony‐forming units (CFUs) per g of fish sample on plate counting agar in Petri dishes, as described by NMKL (2013).

### 2.6 Proximate composition

Water content (g water/100 g) was determined by the weight loss from a 5 g sample of minced fillet during drying in an oven at 104 ± 1°C for 4 h, according to ISO (International Organization for Standardization) (1999). The sodium chloride (NaCl) content (g salt/100 g) was measured using the Volhard titration method (AOAC, 2000). The Bligh and Dyer (1959) method was used to extract the total lipids of all mackerel samples (TL, g of lipid/100 g). The lipid extracts were stored at −80°C until further analysis. Determination of the total nitrogen content was performed using the Kjeldahl method (ISO, 2009). The nitrogen content was multiplied by a conversion factor of 6.25 to estimate the percentage of crude protein in the samples (g of protein/100 g).

### 2.7 Lipid oxidation products

Lipid oxidation was evaluated by the assessment of the primary (lipid hydroperoxide—(PV)) and secondary (thiobarbituric acid‐reactive substances—TBARS) oxidation products. PV and TBARS were assessed in triplicate from minced samples. PV was measured in the muscles according to a modified ferric thiocyanate method (Shantha & Decker, 1994), and the results were expressed as micromole (μmol) lipid hydroperoxide per g of muscle. TBARS were measured using a modified method described by Lemon (1975) and calculated based on a standard curve prepared with 1,1,3,3‐tetraethoxypropane (TEP) and expressed as micromole (μmol) malondialdehyde bis (diethyl acetal) (MDA) per g of sample.

### 2.8 Hydrolysis products

The extent of lipid hydrolysis was assessed by the content of free fatty acids (FFAs) and phospholipids (PLs). FFA was determined according to the Lowry and Tinsley (1976) method with modifications described by Bernárdez et al. (2005). This method is based on a complex formation with cupric acetate‐pyrimidine, followed by an absorbance reading at 710 nm (UV‐1800 spectrophotometer, Shimadzu, Kyoto, Japan). The concentration of FFA was calculated using an oleic acid standard curve in a 0–20 μmol range, and the results were expressed as g of FFA per 100 g of lipid. The PL content of the fish muscle was determined based on the total lipid (TL) extracts using a colorimetric method, based on the complex formation of phospholipids and ammonium ferrothiocyanate (Stewart, 1980), followed by absorbance reading of the resultant solutions at 488 nm (UV‐1800 spectrophotometer, Shimadzu, Kyoto, Japan). A standard curve was prepared with phosphatidylcholine in chloroform (5–50 μg/mL), and the results were expressed as g of PL per 100 g of lipid.

### 2.9 Instrumental texture profile and color analyses

A compression test was used to estimate the hardness of the mackerel fillets using a TA.HDplus Texture Analyser (Stable Micro Systems, Haslemere, Surrey, UK) with a flat‐ended aluminum cylindrical probe (20 mm diameter) and the following settings: 2 mm/s pretest speed, 1 mm/s test speed, 10 mm/s posttest speed, and 5 g trigger force. A CR‐300 Chroma Meter (Minolta Camera Co. Ltd. Osaka, Japan) was used to measure the color of six fillets on each sampling occasion using the CIE L*a*b* scale. Both parameters were evaluated at three locations on the light muscle of the deep‐skinned side of the fillets, two on the loin, and one on the middle, of three fillets per sample.

### 2.10 Sensory analysis

Generic Descriptive Analysis (GDA), as introduced by Lawless and Heymann (2010), was conducted on each sampling occasion to assess changes in sensory attributes throughout the period of chilled storage. Highly experienced panelists underwent training sessions according to international standards (ISO, 2014) to enhance their sensory proficiency in smoked fish, ensuring their ability to deliver reliable assessments across the different chilled storage times of the smoked fillets. During the training sessions, each panelist was guided to identify and assess pertinent descriptive sensory attributes for the final products, emphasizing key aspects, such as flavor, odor, and texture. A final collective panel discussion aimed at standardizing definitions for each of these attributes took place during these training sessions.

Twenty‐one attributes, describing odor (7), flavor (9), and texture (5), were assessed by the sensory panel consisting of 6–11 panelists, depending on the sampling occasion. Around 20–30 g pieces were cut transversally from the middle part of the fillets, placed in aluminum trays precoded with random 3‐digit numbers, then covered with a plastic lid, and presented to members of the panel. Samples were evaluated in blind duplicates by each panelist, in a random order. Panelists marked their sensory scores on a 15‐cm unstructured line scale, and the linear placement of the mark was converted to numbers from 0 to 100 for data analysis purposes (Table S1).

### 2.11 Statistical analyses

Microsoft Office Excel 2016 (Microsoft Inc. Redmond, WA, USA) and SigmaPlot 12.0 (Dundas Software Ltd., GmbH, Germany) were used for statistical analyses. One‐way analysis of variance (ANOVA) and Duncan's multiple comparison test were used to assess differences between means.

For the sensory data, statistical analyses were carried out using the NCSS 2000 statistical program (NCSS, Utah, USA) program. Duncan's post hoc test, an accepted method for sensory statistical data handling, was applied to evaluate changes occurring over time during the storage of different treatments. PanelCheck V1.4.0 software (Nofima, Tromsø, Norway) was used to monitor the panel's performance, and the FIZZ's software (version 2.51C, 1994–2018, Biosystémes) was used to collect the sensory data. Statistical significance was defined as *p* < .05 for all statistical analyses.

## 3 RESULTS AND DISCUSSION

### 3.1 Microbiological quality of raw materials and smoked mackerel

**FIGURE 2:**
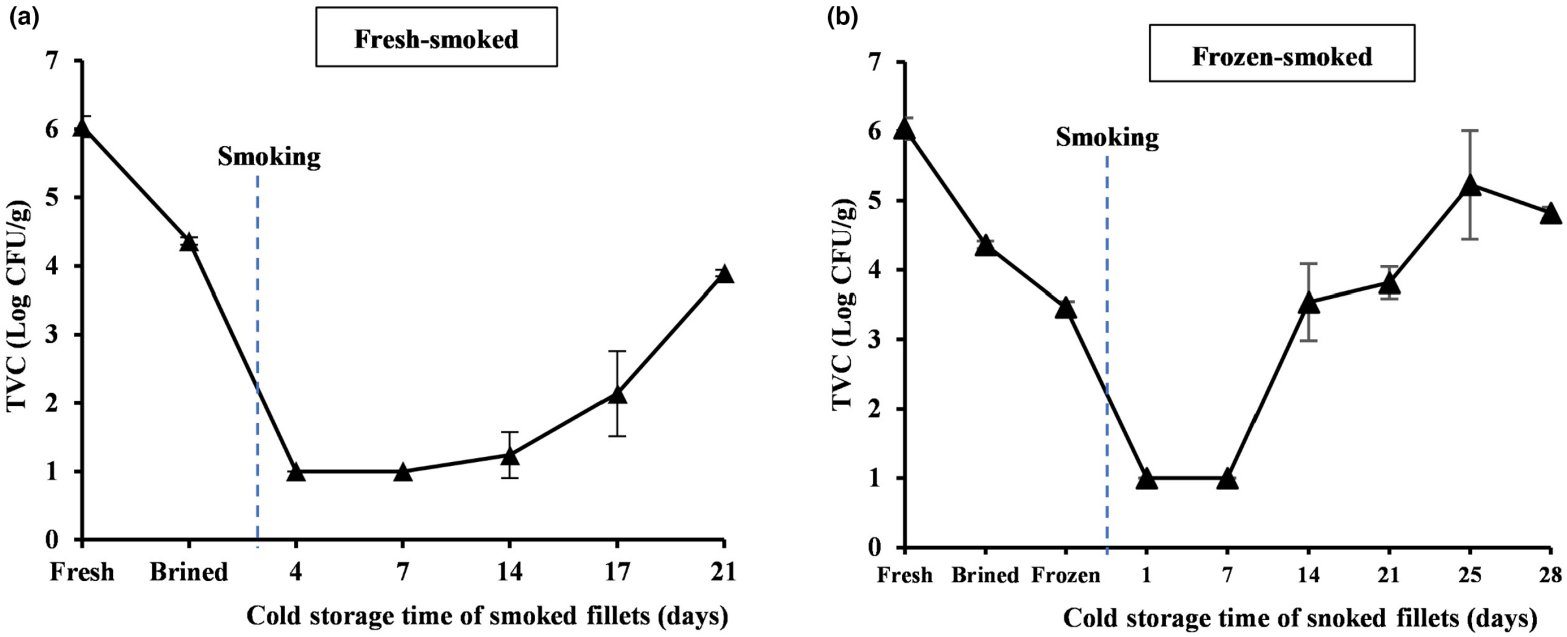
Bacterial growth in fresh‐smoked (a) and frozen–thawed and smoked (b) mackerel fillets stored for up to 28 days at 1 ± 0.6°C. The blue vertical line indicates the timing of the hot‐smoking step within the process.

The highest total aerobic viable counts (6 log CFU/g) were obtained in the fresh mackerel (Figure 2). However, a drastic reduction in TVC occurred after brining (*p* < .05), which decreased even further below the detection threshold of 1 log CFU/g after smoking (*p* < .05), indicating that both brining and hot‐smoking were highly efficient in reducing the microbial load in the fillets. TVC remained stable during the first 7 days of chilled storage both for fresh‐smoked and frozen–thawed fillets (Figure 2a,b), whereafter the values increased with prolonged storage. Despite the significant increase from day 7 to the end of storage (*p* < .05), the values remained below the consumption threshold of 5 log CFU/g, commonly used for smoked fish (Cyprian et al., 2015), for at least 21 days of chilled storage for fresh‐smoked fillets, and at least 28 days for the frozen–thawed smoked fillets.

*Listeria monocytogenes* was present in the fresh raw material but was not detected in any other samples analyzed. *L. monocytogenes* is part of the microbiota regularly found on surfaces in food processing plants despite routine cleaning and disinfection, and can survive and persist in such environments (Fagerlund et al., 2017). Therefore, a high risk exists of cross‐contamination of the fresh raw material by microbial surface‐associated communities (biofilms) (Reynisson et al., 2009) during the skin removal stage, before immersing the fillets in the brine. Smoked fish represents a suitable environment for *L. monocytogenes* (Kolodziejska et al., 2002), as is also the case for other ready‐to‐eat (RTE) food products (Kuzmanović et al., 2011). The ability of *L. monocytogenes* to persist and grow in refrigerated RTE products is due to its psychotropic character and distribution (Garrido et al., 2011). In the current study, however, a complete elimination of this pathogen was observed immediately after brining and washing. The absence of *L. monocytogenes* in six species of smoked fish, including Atlantic mackerel, has also been reported by Anihouvi et al. (2019). A lower TVC in smoked rainbow trout fillets after brining was also observed by Cheng et al. (2023a), where a higher salt solution showed a significantly greater effect, which was ascribed to the inhibitory effect of increased osmotic pressure on the propagation of microorganisms.

### 3.2 Proximate composition of fresh and smoked mackerel

**FIGURE 3:**
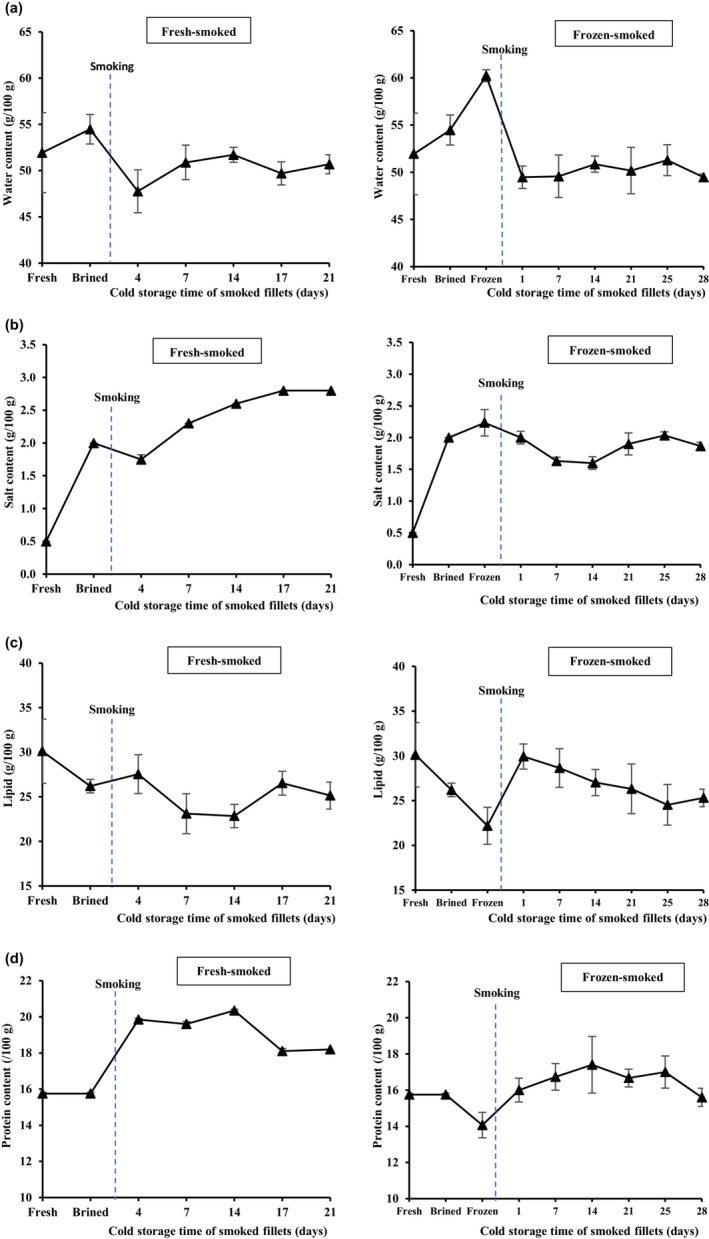
Water (a), salt (b), lipid (c), and protein (d) contents of fresh‐smoked (left) and frozen–thawed and smoked (right) mackerel fillets stored for up to 28 days at 1 ± 0.6°C. The blue vertical line indicates the timing of the hot‐smoking step within the process.

The fresh raw material had a water content of 51.9 ± 4.3 g, salt content of 0.5 ± 0.0 g, lipid content of 30.1 ± 3.6 g, and protein content of 15.8 ± 0.1 g per 100 g of sample (Figure 3). These values are in line with earlier studies on Atlantic mackerel and similar pelagic fish species (Murray & Burt, 2001; Romotowska, Karlsdóttir, et al., 2016a; Sveinsdóttir et al., 2020). Lipid content greater than 20% has been observed in several species after a period of intensive feeding (Murray and Burt 2001). High lipid content poses a risk to the stability of fish products, especially in well‐fed Atlantic mackerel, which is high in polyunsaturated fatty acids (PUFAs) and, therefore, prone to lipid oxidation (Cyprian et al., 2015; Stolyhwo et al., 2006).

During the brining step, both water and salt contents increased, the latter being significant as compared to the fresh raw material (*p* < .05). However, no changes in the protein content were observed during the brining of the fillets (*p* > .05) (Figure 3a,b,d). Water and salt diffusion mechanisms depend on the method of salting and the salt concentrations applied, as well as on the quality, chemical composition, and protein status of the raw material (Arason et al., 2014). The diffusion of salt and water during brining may also be affected by the lipid content and distribution in the raw material (Gallart‐Jornet, Rustad, et al., 2007a; Gallart‐Jornet, Barat, et al., 2007b). A significant decrease in the lipid content after brining (*p* < .05) (Figure 3c) can be explained by the proportional increase of water (Aubourg et al., 2005; Cardinal et al., 2004). Salting exerts an osmotic effect on fish flesh, and as such oil and other liquid or dissolved constituents may be lost from fatty fish during brining (Bligh et al., 1988). Frozen storage of the brined fillets for 9 months led to a significant decrease in protein content, along with significant increases in water and salt contents (*p* < .05). Frozen storage of freshwater (Malik et al., 2021) and marine (Foruzani et al., 2015; Shamsan et al., 2019) fish species has been found to reduce the protein content of fish muscle. Protein and other biochemical components of the fish flesh are known to undergo chemical and physical changes during frozen storage. Frozen storage can lead to protein denaturation, which reduces the amount of soluble proteins (Puke & Galoburda, 2020). The observed decrease in protein content suggests a decrease in the extractability of the proteins and protein denaturation, caused by freezing and thawing (Abraha et al., 2018; Sigurgisladottir et al., 2000) or frozen storage (Puke & Galoburda, 2020).

Hot‐smoking led to a decrease in water content, both in fresh‐smoked (*p* > .05) and frozen–thawed smoked (*p* < .05) mackerel. This can be explained by the partial drying of the muscle, and by heat‐induced losses in the ability of the muscle to retain liquid (Cyprian et al., 2015; Sampels, 2015). Large variations in water content were observed during chilled storage, as evidenced by the large standard deviations (Table S2). This variation observed in water content in the smoked fillets probably reflected differences in the size, shape, and composition of the fillets, rather than storage effects. A decreasing trend in water content has also been reported for smoked skinless rainbow trout (*Oncorhynchus mykiss*) fillets during cold storage, with increased variation among fillets salted in different brine concentrations by Cheng et al. (2023a). The average water content of the smoked fillets of both groups, around 50 g/100 g, meets the industrial specifications for “smoked finished products,” which recommend a water content below 65 g/100 g for this product category (Cardinal et al., 2001). Low water content inhibits microbial growth and improves both sensory stability and shelf‐life (Cardinal et al., 2004). In this study, the salt content increased during the cold storage in the fresh‐smoked mackerel fillets, which agrees with the results reported by Cheng et al. (2023a). As no significant changes were observed in the water content during chilled storage of the fillets, the increase in salt content does not appear to be affected by changes in water retention, but rather by the proportional loss of lipids during the processing and storage of the fresh‐smoked fillets (Figure 3c). Although a similar increase was observed in the salt content during brining prior to freezing, the frozen fillets seemed to be more sensitive toward water loss during the smoking step. This led to smaller changes in the salt content during freezing and smoking, and a fluctuating salt content throughout the cold storage. A salt content of 3% is enough to inhibit the growth of any food poisoning organism, and for smoked fish, such as mackerel, this is especially important for the inhibition of *Clostridium botulinum* and *Listeria monocytogenes* (Bhuiyan et al., 1986), while an acceptable salty flavor is provided to the smoked products (Cyprian et al., 2015). In this study, the average salt content for smoked mackerel was 2.5 ± 0.0 g/100 g muscle in the fresh‐smoked fillets, which is within the salinity range of 2%–6% of commercially available smoked products, as stated by Cheng et al. (2023a), and should be high enough to effectively inhibit the growth of most spoilage and pathogenic microorganisms (Goulas & Kontominas, 2005; Leroi & Joffraud, 2000). After brining and the subsequent steps, the presence of *L. monocytogenes* was not detected in the mackerel fillets. The incidence of *L. monocytogenes* has been reported to be lower in heat‐treated and cured fish products (4%–12%) than in cold‐smoked fish (11%–60%), with a reduced prevalence after processing (2.7%) than during the retail stage (25%) (Cheng et al., 2023a; Kolodziejska et al., 2002). For the frozen–thawed smoked fillets, however, the average salt content was 1.8 ± 0.1 g/100 g, which might explain the slightly faster microbial TVC growth observed during cold storage (Figure 2).

No differences were observed in the lipid content of fresh‐smoked fillets due to the hot‐smoking (*p* > .05), while the frozen–thawed smoked fillets showed a marked increase in lipid content following the smoking process (*p* < .05). In both cases, the lipid content decreased throughout the chilled storage, although with some fluctuations. Nevertheless, the overall changes in lipid content were not significant (*p* > .05), in agreement with the observations of Cyprian et al. (2015). In addition to the wide variation in the lipid content and composition of Atlantic mackerel related to seasonal and geographical variation (Romotowska, Karlsdóttir, et al., 2016c), the distribution of lipids may also vary among different parts of the fish (Ackman & Gunnlaugsdóttir, 1992; Sveinsdóttir et al., 2021). The subdermal fat layer can constitute up to half of the total lipids in Atlantic mackerel (Ackman & Gunnlaugsdóttir, 1992). Removal of the subcutaneous layer of dark muscle may improve lipid stability (Dang et al., 2018; Sveinsdóttir et al., 2021).

Hot‐smoking increased the relative protein content compared to the fresh, brined, and frozen samples (*p* < .05), which can be explained mainly by the relative decrease in water content during processing (Kiczorowska et al., 2019). Protein and amino acid denaturation can occur during the smoking of fish, depending on the length and temperature of the heat treatment, causing physical and chemical changes in the protein properties and a reduction in the biological availability of proteins (Abraha et al., 2018).

### 3.3 Lipid oxidation products

**FIGURE 4:**
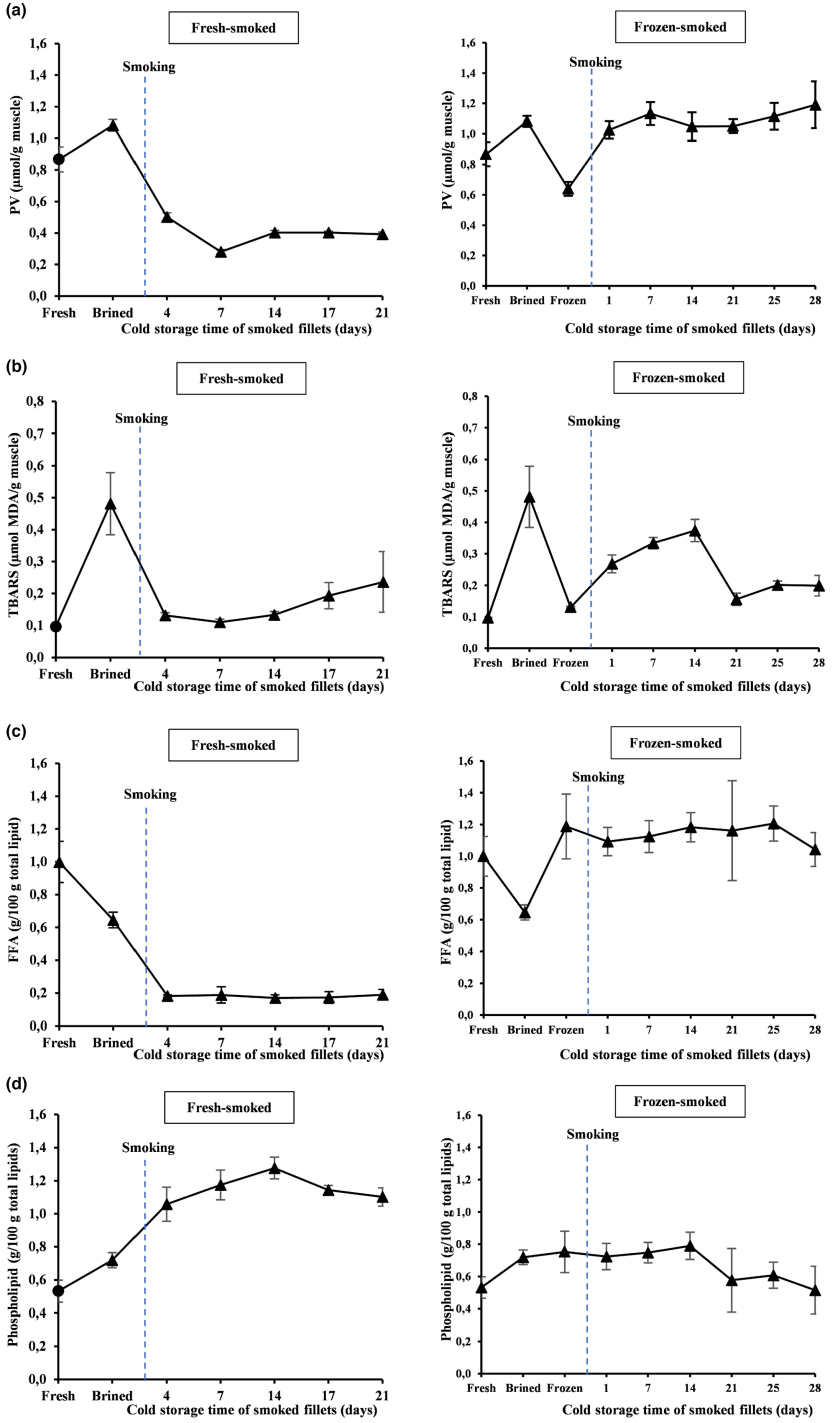
(a) Peroxide value (μmol/g muscle), (b) thiobarbituric acid‐reactive substances (μmol MDA/g muscle), (c) free fatty acid formation (g/100 g of total lipid), and (d) phospholipid content (g/100 g of total lipid) of fresh‐smoked (left) and frozen–thawed smoked (right) mackerel fillets during chilled storage. The blue vertical line indicates the timing of the hot‐smoking step within the process.

Lipid hydroperoxide (PV) and thiobarbituric acid‐reactive substances (TBARS) were used as a measure of primary and secondary lipid oxidation. The initial PV and TBARS concentrations in the raw mackerel were 0.9 ± 0.1 μmol/g muscle and 0.1 ± 0.0 μmol MDA/g muscle, respectively, indicating good raw material quality. Both parameters increased significantly during brining (Figure 4). Enhanced oxidation in the brined samples can be attributed to the prooxidative effect of sodium chloride (Bienkiewicz, Tokarczyk, Czerniejewska–Surm, & Suryn, 2019), which then intensifies the prooxidant effects of chelated iron ions in the fish muscle (Aubourg & Ugliano, 2002).

Lipid oxidation products were effectively decreased when smoking fresh fillets (*p* < .05), which can partially be explained by the interaction between the lipids in the muscle with the phenolic constituents of the smoke, which has well‐known antioxidant properties (Bienkiewicz et al., 2019; Stolyhwo et al., 2006). Unlike the fresh‐smoked fillets, the frozen–thawed smoked fillets showed a marked increase in lipid oxidation products (*p* < .05), probably as a consequence of lipid oxidation during freezing and 9 months of frozen storage (Puke & Galoburda, 2020).

A small but significant decrease was observed in PV during the chilled storage (*p* < .05), while the TBARS values increased slowly with storage time in the fresh‐smoked fillets. The opposite was observed in the frozen–thawed smoked fillets. Changes in PV and TBARS during chilled storage are likely to relate to the different decomposition rates of primary oxidation products toward secondary oxidation (Domínguez et al., 2019). However, the overall concentrations of oxidation products were low in both cases, indicating that the deep‐skinned, smoked, and vacuum‐packed mackerel fillets exhibited high lipid stability during cold storage for at least 28 days. Several factors contribute to storage stability. Studies have shown the benefits of vacuum‐packaging on storage stability and prolonged shelf‐life of fish products compared to iced storage, especially in fatty fish such as mackerel (Goulas & Kontominas, 2007) and herring (Özogul et al., 2000). Another crucial element that should be considered is the removal of the subdermal dark muscle through deep‐skinning, which has higher lipid content and higher concentrations of known prooxidants than the remaining light muscle, in addition to containing large amounts of PUFAs that are easily oxidized (Dang et al., 2017; Sveinsdóttir et al., 2021). The stability of the smoked mackerel fillets could be related to the removal of the dark muscle, which agrees with the observations by Dang et al. (2017), who showed that frozen storage stability of herring fillets could be increased by removing the dark muscle with deep‐skinning.

### 3.4 Lipid hydrolysis products

Free fatty acid and phospholipid contents were used as indicators of enzymatic lipid hydrolysis. In the present study, all samples had low FFA values (Figure 4c), suggesting only minor enzymatic activity in the fish muscle (Cyprian et al., 2015). The initial amount of FFA in the fresh raw material of 1.0 ± 0.1 g/100 g total lipid can be considered as low (Sveinsdóttir et al., 2020), indicating that the mackerel was of high quality and had been handled correctly between the points of catching and processing (Cyprian et al., 2015). However, FFA formation decreased in both fresh and frozen–thawed fillets during brining, while PL concentration increased after the subsequent hot‐smoking step (Figure 4d).

Free fatty acid content was higher when frozen–thawed fillets were smoked. Although the difference was not significant (*p* > .05), there was an apparent increase in this parameter among frozen–thawed samples with time. This observation leads us to assume that enzymatic hydrolysis takes place during frozen storage (Aubourg & Medina, 1999). A high FFA release during frozen storage of mackerel fillets prior to heat treatment has also been reported by Romotowska, Gudjonsdóttir, et al. (2016). Enzymatic lipid hydrolysis, promoted mainly by lipase enzymes (Romotowska, Gudjonsdóttir, et al., 2016), in the frozen–thawed smoked group, may have enhanced lipid oxidation (Ozogul & Balikci, 2013) throughout the chilled storage (Figure 4a,b). The amount of FFA, however, remained stable in both vacuum‐packed, smoked groups throughout the period of chilled storage.

These results lead to the conclusion that the brining and hot‐smoking had an important inhibitory influence on FFA formation and that the storage conditions (vacuum‐packaging and refrigeration) had a stabilizing effect on enzymatic hydrolysis in the smoked fillets (Cyprian et al., 2015). The lipase enzyme, responsible for FFA formation by lipid hydrolysis (Romotowska, Gudjonsdóttir, et al., 2016), may be inhibited by heating (Karlsdottir et al., 2014). The reduction of FFA in fish fillets after heat treatment is in line with a recent study on smoked Atlantic mackerel fillets (Romotowska, Gudjonsdóttir, et al., 2016). However, FFA has been found to increase progressively in hot‐ and cold‐smoked capelin (*Mallotus villosus*) during refrigerated storage (Cyprian et al., 2015) as well as in hot‐smoked herring (*Clupea harengus*) chilled and stored for 9 days (Tenyang et al., 2017). Lipid hydrolysis was more pronounced in capelin when it had a low fat content, but lipid oxidation became the more dominant degradation process in fatty capelin (Cyprian et al., 2015). This demonstrates that FFA development and evolution during processing and storage is highly influenced by the lipid content and composition of the raw material, together with brining, smoking, and storage conditions.

Phospholipid (PL) content increased during brining, smoking, and chilled storage, being significant after smoking fresh fillets (*p* < .05) but not in the frozen–thawed and smoked fillets (*p* > .05) (Figure 4d). Phospholipids, the main constituent of cell membranes, are sensitive to oxidation due to their high content of unsaturated fatty acids (Domínguez et al., 2019). Heating is likely to affect the integrity of cell membranes, causing lipid degradation (Gray & Pearson, 1987) and subsequent easier detection of PL.

During the chilled storage, an increase in PL content in the smoked samples portrayed a corresponding decrease in total lipids, since PL and TL concentrations are inversely related (Weihrauch & Son, 1983). Despite this trend, the PL values in both treatment groups remained low, ranging from 0.7 to 1.3 g/100 g of total lipids during refrigerated storage, indicating low PL hydrolytic activity. The low hydrolytic FFA activity and content of PL observed during the chilled storage might primarily be attributed to heat‐induced inactivation of endogenous enzymes, namely lipases and phospholipases, involved in lipid hydrolysis.

### 3.5 Effects of brining and hot‐smoking on texture and color

The primary method of evaluating the texture profile in the present study was through sensory analysis, as texture characteristics are complex and perceived differently by different consumers. Nevertheless, hardness, identified as a critical factor influencing seafood product freshness and acceptability (Casas et al., 2006; Coppes et al., 2002), was also employed as a supplementary tool to support the findings of the sensory analysis.

**FIGURE 5:**
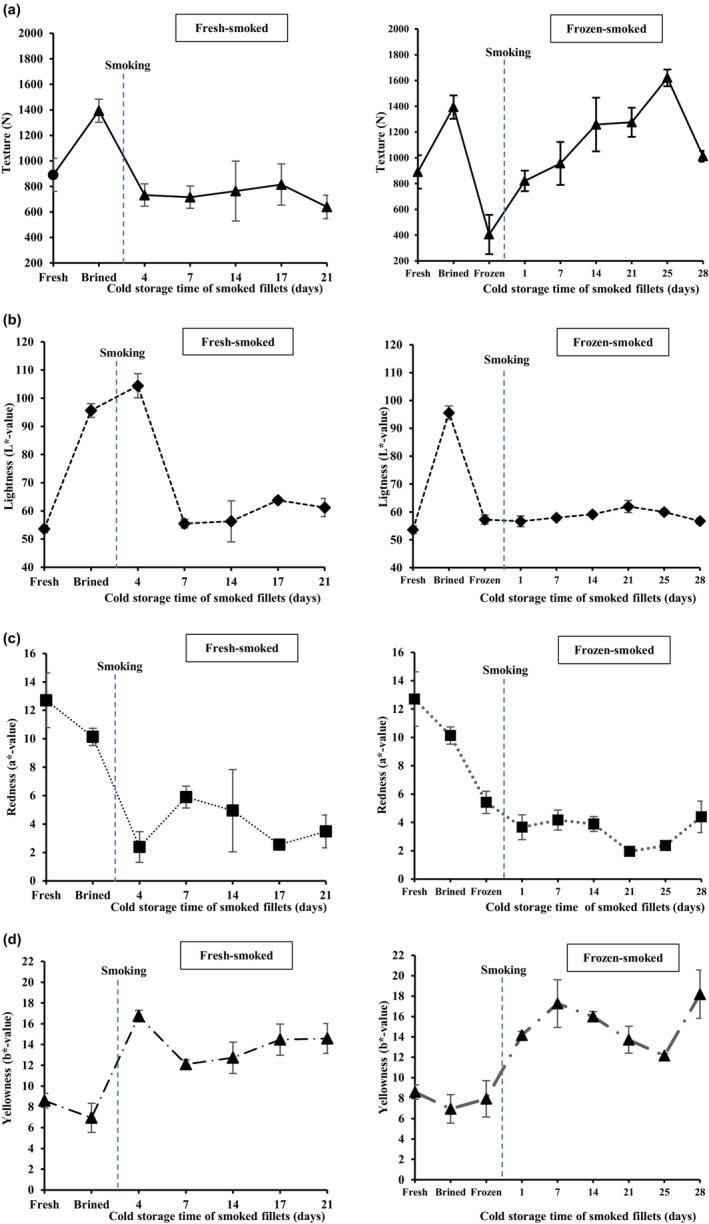
Changes in the texture (a), lightness (b), redness (c), and yellowness (d) of fresh‐smoked (left) and frozen–thawed smoked (right) mackerel fillets during storage at 1 ± 0.6°C for up to 28 days. The blue vertical line indicates the timing of the hot‐smoking step within the process.

Brined fillets had significantly higher maximum resistance force than fresh mackerel (Figure 5a), suggesting that brining was enough to harden the muscle in the fresh skinless fillets (Dhanapal et al., 2013). In this study, the salt content increased from 0.5% to 2.0% after brining. The brine‐induced hardening agrees with those mentioned in earlier studies, where increasing salt concentration and brining time generally resulted in lower elasticity and higher muscle shear force (Gallart‐Jornet, Barat, et al., 2007b; Gallart‐Jornet, Rustad, et al., 2007a; Jittinandana et al., 2002; Thorarinsdottir et al., 2004).

Unlike the frozen–thawed smoked fillets, the fresh‐smoked fillets showed no statistical differences in the hardness after hot‐smoking (*p* > .05). Similar results were also found throughout the refrigerated storage, with some fluctuations in the shear force values registered in both treatments. After smoking and throughout chilled storage, a greater hardness was observed in the frozen–thawed than in the fresh‐smoked fillets (*p* > .05). These findings are consistent with previous research that demonstrated the impact of freezing on the texture and microstructure of smoked fish fillets. A study by Sigurgisladottir et al. (2000), who investigated changes in the microstructure and texture of fresh and frozen–thawed Atlantic salmon during cold smoking (20°C and 30°C), concluded that freezing and subsequent thawing can alter the muscle structure of smoked fillets. A significantly greater hardness was also found at the beginning of chilled storage in Atlantic salmon (*Salmo salar*) fillets frozen–thawed before smoking, and then decreased until the end of a 45‐day storage period (Martinez et al., 2010), while Gallart‐Jornet, Rustad, et al. (2007a) reported a significant reduction in the hardness along with increased cohesiveness during storage. Fish muscle undergoes structural changes during freezing due to the shrinking of muscle fibers resulting from the movement of water into the extracellular spaces (Sigurgisladottir et al., 2000). Protein denaturation may occur during freezing, affecting muscle texture, as proteins are the principal contributors to texture properties (Abraha et al., 2018).

A lighter appearance was observed upon immersing the fillets in a 15% NaCl brine solution, as evidenced by a significant increase in the lightness (L*‐values) (*p* < .05), and simultaneously a reduction in redness (a*‐values) and yellowness (b*‐values) (Figure 5b–d). This may be attributed to increased water absorption during the brining step (Figure 3a), which might have induced changes in the refraction of light on the surface of the fillets (Nguyen et al., 2012). The color and appearance of salted fish products may be improved by brine salting at levels <20% NaCl (Thorarinsdottir et al., 2004).

During smoking, the fish muscle is exposed to the smoke produced during the pyrolysis of wood, which provides heat and chemical compounds, such as phenols and formaldehyde, thus changing the product's color (Abraha et al., 2018). The color intensity relies greatly on the phenol content that is deposited in the flesh, where higher phenol content has been linked to the formation of an appealing golden‐brown color (Belichovska et al., 2019). A noticeable and desirable golden‐brown color (i.e., a more intense yellowish tone and less red tone) was observed in all samples. This may be a consequence of the high temperature applied during the hot‐smoking, which may have increased the deposition of smoke compounds (Arason et al., 2014; Cardinal et al., 2001).

Although the same pattern in color changes was observed in both the fresh and frozen–thawed fillets during storage, these changes were a little more pronounced in the frozen–thawed smoked fillets. Freezing and thawing may damage the structure of the native protein, becoming more prone to further reactions (Timberg et al., 2014). According to Martinez et al., 2010, freezing before smoking negatively affects the color intensity of fish flesh. Overall, however, these color variations due to the different processing methods did not greatly affect the color of smoked fillets.

### 3.6 Effects of hot‐smoking on sensory attributes

Smoking imparted an intense smoky odor and ‐flavor to both fresh and frozen–thawed fillets, which increased significantly in the frozen–thawed smoked fillets during chilled storage (*p* < .05) (Table 1). The development of a smoky odor and flavor after hot‐smoking observed in this study agrees well with Goulas and Kontominas (2005), who ascribed their results to the temperature and contact time between the fish fillets and the wood smoke. During hot‐smoking, the characteristic “smoke” odor and flavor could have been attained through the assimilation of not only various volatile compounds, most importantly phenols, but also esters, ethers, alcohols, and ketones, released from the combustion of wood (Rana et al., 2021). Phenols also have preservative and antioxidative effects (Goulas & Kontominas, 2005; Stolyhwo et al., 2006). The rich smoky odor may have been influenced by the high temperature during smoking, which reached about 80°C in the smoke chamber at the final stage of the process. Sérot et al. (2004) reported increased deposition of phenolic compounds with time when temperature increased, which suggested that this may have been caused by an increase of phenolic compounds in the vapor phase at high temperatures. The high temperature allows the compounds of higher molecular weight involved in the smoke effect to remain in the vapor phase, resulting in their greater deposition in the products (Cardinal et al., 2006). Sensory evaluation of both fresh‐ and frozen–thawed smoked fillets showed that the hot‐smoking resulted in a slightly salty flavor with traces of metallic notes. Flavor parameters indicating deterioration (bitter, rancid, etc.) were barely noticeable in any of the samples. It is well known that smoking can improve the acceptability of fish by providing pleasant flavors, in addition to increasing shelf‐life (Huang et al., 2019). The salty flavor was only found to be statistically more intense in fresh‐smoked fillets on day 21 compared to day 17, although the salt content was not significantly different between these 2 days (Figure 3b).

**TABLE 1:** Changes in sensory attributes of deep‐skinned fresh and frozen–thawed smoked vacuum‐packed mackerel fillets, stored at 1 ± 0.6°C for up to 28 days.

Sensory attribute	Chilled storage duration (days) 1 ± 0.6°C											
	Fresh‐smoked deep‐skinned fillets					Frozen–thawed smoked deep‐skinned fillets						
	7	14	17	21	*p*‐Value	1	7	14	21	25	28	*p*‐Value
ODOR												
Smoke	54	55	51	57	0.828	47^a^	37^c^	40^bc^	36^c^	46^ab^	48^a^	0.000
Butyric acid	1	0	1	1	0.736	1	3	2	2	2	3	0.644
Rancid	2	2	1	2	0.953	4	3	4	4	3	3	0.853
Spoilage sour	1	1	1	1	0.918	1	1	2	1	1	1	0.119
TMA	1	0	1	1	0.732	1	1	1	1	1	1	0.890
Spoilage odor	1	1	1	1	0.885	1	3	3	3	1	2	0.125
Frozen storage						1	4	3	4	4	2	0.053
FLAVOR												
Smoke	55	55	53	55	0.966	47	41^b^	42^b^	40^b^	45	52^a^	0.001
Metallic	25	23	27	16	0.341	19	18	18	18	20	19	0.865
Salty	35	42	26^b^	49^a^	0.061	34	38	31	35	33	37	0.442
Bitter	11	18	12	16	0.295	9	13	14	13	12	13	0.375
Rancid	1	1	1	3	0.257	3	7	6	7	4	4	0.177
Spoilage sour	1	1	1	1	0.628	1	4	2	4	3	1	0.380
TMA	1	0	1	1	0.581	0	2	1	3	2	1	0.310
Spoilage flavor	1	1	1	1	0.783	4	8	6	8	4	5	0.295
Frozen storage						3	7	6	7	4	4	0.236
TEXTURE												
Soft	71	71	77	81	0.085	66	68	64	70	65	68	0.497
Juicy	67	68	68^b^	79^a^	0.056	65	64	62	63	65	65	0.608
Tender	73	72	77	81	0.245	69	68	64	69	64	70	0.158
Mushy	51	46	43	51	0.750	33	28	23	31	27	33	0.110
Fat in mouth	29	41	40	39	0.160	28	31	29	30	27	29	0.900

*Note*: Lowercase letters (a–c) indicate significant statistical differences (*p* < .05) between samples during storage in the fresh‐smoked fillets and frozen‐smoked fillets, respectively.

All smoked samples had relatively high average scores for tenderness, softness, and juiciness, while lower scores were attributed to mushy and fat‐in‐mouth sensations. The juiciness of the fresh‐smoked mackerel fillets, however, was significantly enhanced during the last week of storage (*p* < .05), whereas other textural attributes did not change during the storage period (*p* > .05). This may indicate that the storage conditions did not have a major influence on the texture of fresh‐smoked fillets, which correlates well with the results of the instrumental texture analysis.

The sensory evaluation was, in general, in good agreement with the assessed physicochemical quality and microbiological indices. The sensory scores are indicative of low microbial activity during storage (Figure 2). Typically, microbial activity during chilled storage is the main cause of a reduction in the shelf‐life of processed seafood (Kolodziejska et al., 2002). Likewise, the low lipid oxidation development over the storage period, as evidenced by PV and TBARS measurements (Figure 4a,b), is in agreement with the barely noticeable sensory deterioration attributes in the smoked fillets.

In general, slightly lower freshness scores (smoky odor and flavor, as well as texture scores) were obtained for frozen–thawed smoked fillets, along with slightly higher scores for spoilage indicators (e.g., rancidity, spoilage sour, and frozen storage odors and flavors) than those observed in the fresh‐smoked fillets. Although freezing/thawing and/or long‐term frozen storage may have led to some minor changes in the sensory quality of smoked end products, the sensory scores of both the fresh and frozen/thawed smoked fillets remained within acceptable limits for the duration of storage. Puke and Galoburda (2020) reported that smoked sprat (*Sprattus sprattus balticus*) made from frozen/thawed raw material tended to be sour and developed a rancid taste more rapidly than when using fresh sprat. Freezing before smoking had a negative effect on smoke odor and color intensity, as well as textural properties (Martinez et al. 2010). Protein denaturation, which correlates strongly with loss of sensory quality, has been shown to cause loss of quality in frozen fish (Puke & Galoburda, 2020). The results of this study demonstrate, therefore, how important proper handling and deep‐skinning of fillets are for the quality of the final products.

Overall, the results show a high sensorial quality of the smoked mackerel fillets regarding odor, flavor, and texture profiles during the whole experimental storage period of both the fresh‐ and frozen‐smoked fillets. A combination of factors, such as a proper smoking protocol, i.e., controlled time and temperature (Abraha et al., 2018), the concentration of smoke (Belichovska et al., 2019), as well as the type of wood used to produce the smoke (Abraha et al., 2018; Belichovska et al., 2019), may have accounted for these results. The results suggest that the low storage temperature and vacuum‐packing had a preserving effect on the final products, maintaining the desired sensorial characteristics during the tested storage time. Although polycyclic aromatic hydrocarbon (PAH) contamination has been widely addressed recently as one of the main concerns related to smoked fish (Aksun Tümerkan, 2022; Asamoah et al., 2021; Hokkanen et al., 2018; Tiwo et al., 2019), this issue was not addressed in the present study, but should be considered in further product development and process optimization of hot‐smoked Icelandic mackerel.

## 4 CONCLUSIONS

Changes in microbiological, sensory, and physicochemical properties of well‐fed Atlantic mackerel fillets were assessed throughout chilled storage. The fillets were subjected to deep‐skinning, followed by hot‐smoking and vacuum‐packing, either in their fresh state or after being frozen and thawed. Brining and hot‐smoking had a significant positive influence on the microbial quality and safety of the fillets, eliminating *L. monocytogenes*, and reducing significantly the total aerobic flora compared to the initial raw mackerel. Hot‐smoking had a significant effect on the proximate composition of the mackerel fillets, mainly due to heat‐induced water loss. Smoking retarded lipid deterioration of the products over the storage period. The hot‐smoking enhanced the sensory attributes of the mackerel fillets, giving the products an intense and pleasant smoke‐like odor and flavor, and a tender texture. Although the frozen–thawed smoked fillets were shown to be of slightly lower quality and less stable toward lipid and enzymatic degradation, they still maintained acceptable sensory and total viable count values for human consumption for at least 28 days at 1 ± 0.6°C. Producers can thus use frozen materials for the production of hot‐smoked Atlantic mackerel fillets, which allows more flexibility in processing to adjust to raw material availability and processing capacity.

The study demonstrated that deep‐skinned fillets from well‐fed Atlantic mackerel, caught during the summer in Icelandic waters, constitute an excellent raw material, suitable for producing stable, high‐quality smoked products for human consumption, both when sourced fresh and frozen–thawed.

## AUTHOR CONTRIBUTIONS

**Carina Mascarenhas Fernandes:** Conceptualization (lead); data curation (lead); formal analysis (equal); investigation (lead); methodology (equal); software (lead); visualization (equal); writing – original draft (lead); writing – review and editing (equal). **Hildur Inga Sveinsdóttir:** Conceptualization (equal); funding acquisition (equal); methodology (equal); project administration (equal); validation (equal); visualization (equal); writing – review and editing (equal). **Tumi Tómasson:** Conceptualization (equal); funding acquisition (equal); methodology (equal); project administration (equal); validation (equal); visualization (equal); writing – review and editing (equal). **Sigurjon Arason:** Conceptualization (equal); formal analysis (equal); funding acquisition (equal); investigation (equal); methodology (equal); project administration (equal); supervision (equal); validation (equal); writing – review and editing (equal). **María Guðjónsdóttir:** Conceptualization (equal); formal analysis (equal); funding acquisition (lead); investigation (equal); methodology (equal); project administration (lead); supervision (equal); validation (equal); visualization (equal); writing – original draft (equal); writing – review and editing (equal).

## CONFLICT OF INTEREST STATEMENT

The authors declare that they do not have any conflict of interest.

## ETHICAL STATEMENTS

Ethical Review: This study does not involve any human or animal testing.

## INFORMAL CONSENT

Written informed consent was obtained from all study participants.

## Supporting information


Table S1.
Table S2.

## Data Availability

The data that support the findings of this study are available on request from the corresponding author. The data are not publicly available due to privacy or ethical restrictions.
