# Supplementary material for: Influence of hot‐smoking on the stability of fresh and frozen–thawed deep‐skinned Atlantic mackerel fillets during cold storage
Source: Food Sci Nutr. 2024 Apr 8;12(7):4849–64. doi: 10.1002/fsn3.4132 (PMC11266935; doi:10.1002/fsn3.4132)
Supplement: Supplementary file 1 — Table S1. Table S2. [file FSN3-12-4849-s001.docx]

**Table S1.** Sensory attributes, their description, and GDA scale for deep-skinned fresh and frozen-thawed (9 months at -25 °C ± 1.8 °C) smoked mackerel fillets.

| **Sensory attribute** | **Short name** | **Scale** | **Description** |
| --- | --- | --- | --- |
| ODOR | | | |
| Smoke | O-smoke | none \|\| much | Strength of smoke odor in smoked mackerel |
| Butyric acid | O-butyric | none \|\| much | Butyric acid, smelly feet |
| Rancid | O-rancid | none \|\| much | Rancid odor |
| Spoilage sour | O-sour | none \|\| much | Spoilage sour odor |
| TMA | O-TMA | none \|\| much | TMA odor, amine, dried fish |
| Spoilage odor | O-spoilage | none \|\| much | Strength of spoilage odor |
| Frozen storage | O-frozen | none \|\| much | Frozen storage odor, cardboard |
| FLAVOR | | | |
| Smoke | F-smoke | none \|\| much | Strength of smoke flavor in smoked mackerel |
| Metallic | F-metallic | none \|\| much | Metallic flavor |
| Salty | F-salty | none \|\| much | Salty flavor |
| Bitter | F-bitter | none \|\| much | Bitter flavor |
| Rancid | F-rancid | none \|\| much | Rancid flavor |
| Spoilage sour | F-sour | none \|\| much | Spoilage sour flavor |
| TMA | F-TMA | none \|\| much | TMA flavor, amine, dried fish |
| Spoilage flavor | F-spoilage | none \|\| much | Strength of spoilage flavor |
| Frozen storage | F-frozen | none \|\| much | Frozen storage flavor, cardboard |
| TEXTURE | | | |
| Soft | T-soft | firm \|\| soft | Softness in first bite |
| Juicy | T-juicy | dry \|\| juicy | Dry: draws liquid from mouth. Juicy: releases liquid when chewing |
| Tender | T-tender | tough \|\| tender | Tenderness when chewing a few times |
| Mushy | T-mushy | none \|\| much | Mushy, porridge-like texture when chewing |
| Fat in mouth | T-fat | none \|\| much | Amount of fat in the mouth when chewing |

**Table S2.** Proximate composition of fresh, brined, frozen-thawed (9 months at -25 ± 1.8 °C), and smoked mackerel fillets during chilled storage (1 ± 0.6 °C).

| **Raw material** | | **Proximate composition (%)** | | | |
| --- | --- | --- | --- | --- | --- |
|  |  | **Water content** | **Salt content** | **Protein content** | **Lipid content** |
|  |  | **X ± SD^†^** | | | |
| Fresh fillets | | 51.9 ± 4.32**^A^** | 0.5 ± 0.00**^A^** | 15.8 ± 0.07**^A^** | 30.1 ± 3.60**^A^** |
| Brined fillets | | 54.5 ± 1.59**^A^** | 2.0 ± 0.00**^B^** | 15.8 ± 0.07**^A^** | 26.2 ± 0.76**^A^** |
| Frozen-thawed fillets | | 60.2 ± 0.63**^B^** | 2.2 ± 0.21**^C^** | 14.1 ± 0.70**^B^** | 22.2 ± 2.06**^B^** |
| Fresh-smoked fillets | Day 4 | 47.8 ± 2.32**^a^** | 1.8 ± 0.07**^a^** | 19.9 ± 0.07**^a^** | 27.5 ± 2.17**^a^** |
|  | Day 7 | 50.9 ± 1.86**^a^** | 2.3 ± 0.00**^b^** | 19.6 ± 0.14**^b^** | 23.1 ± 2.24**^b^** |
|  | Day 14 | 51.7 ± 0.81**^a^** | 2.6 ± 0.00**^c^** | 20.4 ± 0.07**^c^** | 22.8 ± 1.30**^b^** |
|  | Day 17 | 49.7 ± 1.24**^a^** | 2.8 ± 0.00**^d^** | 18.1 ± 0.14**^d^** | 26.5 ± 1.34**^ab^** |
|  | Day 21 | 50.7 ± 1.03**^a^** | 2.8 ± 0.00**^d^** | 18.2 ± 0.00**^d^** | 25.1 ± 1.51**^ab^** |
| Frozen-thawed- smoked fillets | Day 1 | 49.5 ± 1.19**^a^** | 2.0 ± 0.10**^a^** | 16.0 ± 0.66**^a^** | 29.9 ± 1.40**^a^** |
|  | Day 7 | 49.6 ± 2.25**^a^** | 1.6 ± 0.06**^b^** | 16.7 ± 0.74**^a^** | 28.6 ± 2.16**^ab^** |
|  | Day 14 | 50.9 ± 0.85**^a^** | 1.6 ± 0.10**^b^** | 17.4 ± 1.56**^ab^** | 27.0 ± 1.46**^abc^** |
|  | Day 21 | 50.2 ± 2.46**^a^** | 1.9 ± 0.17**^a^** | 16.7 ± 0.49**^a^** | 26.3 ± 2.78**^abc^** |
|  | Day 25 | 51.3 ± 1.64**^a^** | 2.0 ± 0.06**^a^** | 17.0 ± 0.89**^a^** | 24.5 ± 2.27**^c^** |
|  | Day 28 | 49.5 ± 0.35**^a^** | 1.9 ± 0.06**^a^** | 15.6 ± 0.50**^ac^** | 25.3 ± 0.98**^bc^** |

**^†^**Average ± standard deviation (SD).

Different capital superscript letters (A, B, C) indicate a significant difference at level *p* < 0.05 between fresh, brined, and frozen-thawed fillets based on Duncan’s multiple comparison tests.

Different lowercase superscript letters (a, b, c, d) indicate a significant difference at level *p* < 0.05 between the smoked fillets from the same treatment (fresh smoked/frozen-thawed smoked)/column during chilled storage based on Duncan’s multiple comparison tests.
